# Revealing tumor microenvironmental heterogeneity and prognostic value in angioimmunoblastic T-cell lymphoma via spatial transcriptome sequencing

**DOI:** 10.1038/s41419-025-08212-9

**Published:** 2026-01-09

**Authors:** Xiang Zhang, Yong Sun, Duo Wu, Fang Yu, Hanjin Yang, Xingnong Ye, Juying Wei, Xuewu Zhang, Yanan Zhu, Yunfei Lv, Zijing Xu, Yuxiang Chen, Hongyan Tong, Jie Jin, Xiaofei Ye, Wenjuan Yu

**Affiliations:** 1 Department of Hematology, The First Affiliated Hospital, Zhejiang University School of Medicine, Hangzhou, Zhejiang China; 2 Kindstar Global Precision Medicine Institute, Wuhan, Hubei China; 3 Department of Pathology, The First Affiliated Hospital, Zhejiang University School of Medicine, Hangzhou, Zhejiang China

**Keywords:** T-cell lymphoma, Cancer microenvironment

## Abstract

Angioimmunoblastic T-cell lymphoma (AITL) represents the second most prevalent subtype of peripheral T-cell lymphoma, characterized by a dismal prognosis. However, a systematic exploration of tumor microenvironment (TME) features and their prognostic significance in AITL remains notably deficient. To address this knowledge gap, we conducted spatial transcriptome sequencing (ST-SEQ) and whole-exome sequencing in four AITLs and two noncancerous lymph nodes for discovery purposes, complemented by immunohistochemistry analyses on 37 AITL cases for validation. We identified 14 ST clusters, including five neoplastic clusters, wherein a global shift in B-cell phenotypes and enrichment of myeloid cells were observed. These findings underscore a hallmark of exacerbated inflammation and immune dysregulation within the neoplastic TME. Among the 4 ST-sequenced AITLs, 3 expressed high *CD40*-*CD40LG* activity, accompanied by the upregulation of immune-suppressive-associated genes, such as *CCL17* and *PDCD1*. Conversely, the remaining patient displayed an uncommon absence of *CD40*-*CD40LG* activity but harbored a phagocytosis-associated tumor-associated macrophage (TAM)-enriched TME, which correlated with significantly reduced relapse rates and longer event-free survival (EFS), highlighting the critical value of precise TME stratification in tailoring AITL therapeutic strategies. Finally, trajectory analysis unveiled a distinct trajectory of molecular evolution within this TME landscape. Collectively, our findings illuminate the heterogeneity and prognostic implications of the TME in AITL, providing a robust foundation for the rational design of targeted immunotherapeutic approaches. These insights may substantially advance the development of personalized treatment strategies for AITL patients.

## Introduction

AITL represents the second most common subtype of peripheral T-cell lymphoma [1], characterized by an aggressive clinical course with a five-year overall survival (OS) rate of less than 40% [2]. AITL presents exacerbated inflammatory response and immune dysfunction, predisposing patients to recurrent infections and autoimmunity phenomena. Neoplastic cells of AITL are thought to originate from the monoclonal expansion of follicular helper T cells (TFHs) [3, 4], manifesting as CD4^+^CD8^-^ T-cell receptor (TCR) α/β cells that express CXCL13, CD10, BCL6, PD-1, and ICOS [2, 5–8]. Clinically, AITL is characterized by generalized lymphadenopathy with systemic inflammation/immune-related manifestations, predominantly affecting older adults [2]. The Epstein-Barr virus (EBV) is an oncogenic infectious agent implicated in up to 80% of AITL cases [9, 10]. Currently, CHOP-based regimens remain the frontline treatment for AITL.

The TME of AITL exhibits remarkable heterogeneity. Morphologically, three overlapping histologic patterns have been described: Pattern I (∼20% of cases) preserves lymph node architecture with hyperplastic B-cell follicles [11]. Pattern II (∼30%) is characterized by depleted follicles with concentrically arranged follicular dendritic cells (FDCs). Pattern III (∼50%), the classic AITL histologic pattern, exhibits complete effacement of typical architecture with absent B-cell follicles [12]. This pattern consists of neoplastic TFH cells interspersed with reactive small lymphocytes, histiocytes or epithelioid cells, large B-immunoblasts, eosinophils, and plasma cells, accompanied by a prominent proliferation of high endothelial venules (HEVs), expanded FDC networks, and paracortical B-lymphoid immunoblasts [13–16].

Over the past decade, high-throughput technologies such as genomic and single-cell RNA sequencing (scRNA-SEQ) have enabled in-depth analyses of lymphoma mutagenesis and TME [17, 18]. Genomic sequencing has identified recurrent mutations in *TET2*, *DNMT3A*, *IDH2*, and *RHOA*^*G17V*^ mutations [19–21], implicating epigenetic dysregulation in AITL oncogenesis [22]. scRNA-SEQ revealed that AITL lymphomagenesis is supported by germinal center (GC) B cells via the *CD40*–*CD40LG* axis [23], with a TME characterized by the loss of *CD73* and *CXCR5* expression in B cells and an exhausted phenotype of expanded CD8^+^ T cells [24]. ST-SEQ enables transcriptomic profiling at precise tissue locations, but the TME architecture of AITL in ST resolution remains less investigated [25, 26]. Here, by integrating genomic sequencing, ST-SEQ, and published scRNA-SEQ data, we constructed a comprehensive landscape of the TME in AITL, delineating its heterogeneity and prognostic significance and uncovering its implications for therapeutic strategies.

## Results

### Clinical and genetic background of AITL

**Fig. 1:**
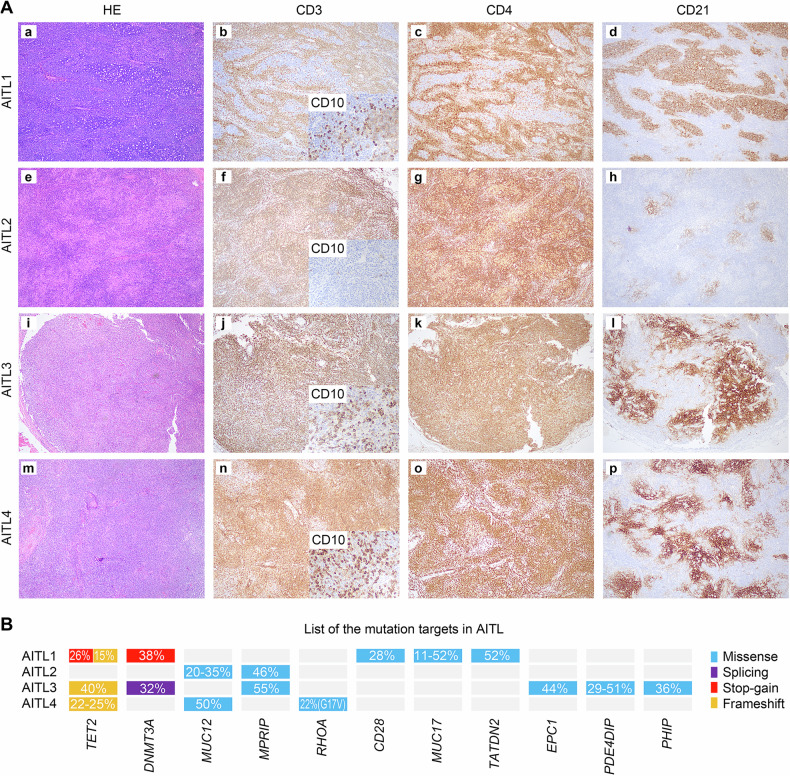
H&E staining, immunostaining, and mutational profile of AITLs. **A** In AITL1, the low-power view (**a**) shows that the contour of lymphoid follicles is serpiginous with atypical lymphoid hyperplasia in the paracortical regions, which are positive for CD3 (**b**), CD10 (B, inset), and CD4 (**c**). CD21 immunostaining (**d**) revealed that follicular dendritic cell networks were mostly preserved. (**e**) In AITL2, the low-power view shows effacement of the lymph node structure, and atypical lymphoid cells are positive for CD3 (**f**) and CD4 (**g**) but not CD10 (f, inset), which intermingle with many pale histiocytic cells with weak positivity for CD4 (**g**). CD21 immunostaining (**h**) revealed mild proliferation of extrafollicular dendritic cells. (**i**) In AITL3, the low-power view shows the effacement of the lymph node structure, and atypical lymphoid cells are positive for CD3 (**j**), CD10 (j, inset), and CD4 (**k**). CD21 immunostaining (**l**) revealed robust proliferation of extrafollicular dendritic cells. (**m**) In AITL4, the low-power view shows the effacement of the lymph node structure, and atypical lymphoid cells are positive for CD3 (**n**), CD10 (n, inset), and CD4 (**o**). CD21 immunostaining (**p**) revealed robust proliferation of extrafollicular dendritic cells. **B** List of mutation targets in AITL.

Tumor samples from 4 AITL patients and inflamed lymph nodes from 2 noncancerous donors (hereinafter referred to as LNs) were collected as the discovery cohort. The average ages of the patients and noncancer donors were 71 years (range: 62–82) and 21 years (range: 17–25), respectively. All patients tested positive for EBV infection and were treated with CHOP-like regimens (Table S1). Histologically, the AITL1 sample exhibited a serpiginous contour of lymphoid follicles, characterized by atypical lymphoid hyperplasia in the paracortical regions, and demonstrated positive immunostaining for CD3, CD4, and CD10. The follicular dendritic cell networks of the AITL1 patient were mainly preserved and CD21-positive, suggesting a histological pattern I. AITL2, AITL3, and AITL4 patients showed effacement of the lymph node structure and atypical lymphoid cells that were CD3- and CD4-positive, suggesting a histological pattern II/III. In contrast to AITL3 and AITL4, which were positive for CD10 and showed widespread proliferation of extrafollicular dendritic cells via CD21 staining, AITL2 was negative for CD10, intermingled with a large number of pale histiocytic cells with weak positivity for CD4, as well as mild proliferation of extrafollicular dendritic cells (Fig. 1A).

Recurrently mutated genes in the four AITLs were identified via whole-exome sequencing (WES) (Tables S2–3), including frequently mutated epigenetic modifier genes such as *TET2* (3/4), *DNMT3A* (2/4), and *RHOA*^*G17V*^ (1/4). Most of these mutations either led to a premature stop codon (stop-gain), generated indels that shifted the reading frame (frameshift), or the recurrent *RHOA*^*G17V*^ mutation, which led to a loss of GTPase activity [27], suggesting the reliability of our mutation calling. Notably, AITL2 did not contain any of these recurrent mutations in the epigenetic modifier genes, indicating that its genetic background related to lymphomagenesis differed from that of the other AITLs. Finally, all the recurrent mutations in our AITL samples had VAFs greater than 20%, indicating that the tumor contents of the AITLs were also greater than 20% (Fig. 1B).

### ST landscape of AITL

**Fig. 2:**
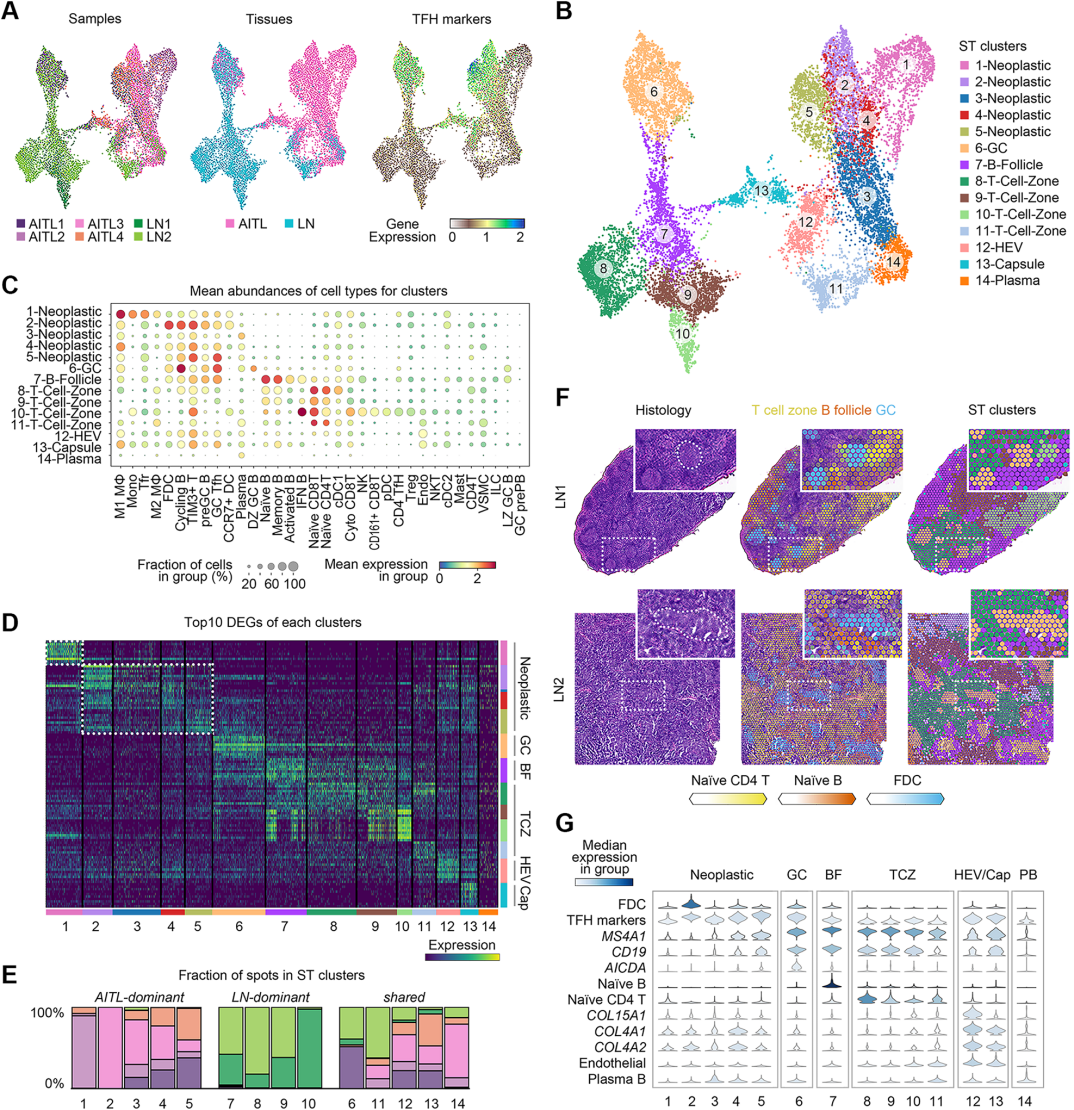
ST landscape and cluster annotation of AITL. **A** UMAP projections of integrated ST spots with information on sample sources, tissue types, and average expression of TFH cell markers, including *CXCL13*, *MME*, *BCL6*, *PDCD1*, and *ICOS*. **B** Annotations of ST clusters. **C** Abundances of the cell populations in the clusters. The abbreviations of the cell types are given in Table S5. **D** Top 10 DEGs for each cluster. The rows represent the top genes. **E** The fraction of spots from each sample for each cluster. **F** Frozen sections with H&E staining, predicted abundances of cell populations, and ST cluster labels in LNs. The clusters in the third column are colored according to the annotation shown in **B**. **G** Selected abundances of cell populations or the expression of genes in each cluster.

ST-SEQ was performed on frozen OCT-embedded tissues from both the AITL and the LN samples. Due to limitations in sample collection, the AITL sections used for ST-SEQ (Fig. S1A) were from the same tissues as the nonadjacent sections shown in Fig.1A. A total of 16,063 qualified ST spots were obtained, with an average of 2677 (1439–4799) spots per sample and an average of 3973 (503–8856) genes per spot (Table S4). After batch effect correction, the spots were integrated and further visualized using uniform manifold approximation and projection (UMAP). The majority of spots from the AITLs and LNs were separated, suggesting a notable difference in cell composition between TMEs of AITLs and noncancerous lymph nodes (Fig. 2A). A total of fourteen ST clusters were identified (Fig. 2B). To determine the abundance of different cell types in the spots, a single-cell dataset of 34 cell populations from human secondary lymphoid organs was projected onto the spots with different scores via a Bayesian model [28] (Fig. 2C and Table S5). Additionally, the DEGs of each ST cluster were identified (Fig. 2D and Table S6).

In Visium ST-SEQ, the diameter of each spot is 55 μm, suggesting that each spot would putatively catch a group rather than a single cell. Thus, neoplastic cell-containing spots could consist of both neoplastic and TME cells. To annotate the clusters containing neoplastic cells (referred to as neoplastic clusters), the sample source composition of each cluster was estimated. Five (clusters 1-5) and four (clusters 7-10) with greater than 95% spots from either AITL or LN, respectively, were identified. The spots from clusters 1-5 also highly expressed TFH cell markers (*CXCL13*, *MME*, *BCL6*, *PDCD1*, and *ICOS)* (Fig. 2A). Therefore, clusters 1-5 were defined as neoplastic clusters, while the remaining clusters 6-14 were defined as nonneoplastic (Fig. 2E). When the spots from only the AITL samples were reclustered, the spots from the neoplastic and nonneoplastic ST clusters remained separated, supporting the robustness of the neoplastic identification (Fig. S2).

Next, to annotate the clusters predominantly composed of spots in LN samples, histological features based on H&E staining and genetic features based on ST-SEQ were investigated. The abundances of naïve CD4 T cells, naïve B cells, and FDCs, as predicted in Fig. 2C were used to represent the GCs, B follicles, and T-cell zones, respectively. By cross-aligning the H&E staining and ST cluster panels, cluster 6 was identified as a GC cluster, which was also supported by the high expression of TFH markers in cluster 6 (Fig. 2A). Cluster 7 was identified as a B follicle cluster, and clusters 8-11 were identified as T-cell zone clusters (Fig. 2F). Finally, for the remaining unannotated clusters 12-14, both clusters 12 and 13 highly expressed collagen genes (*COL15A1*, *COL4A1*, and *COL4A2*) and presented high scores for endothelial cells. Due to its dispersed appearance, cluster 12 was inferred to be HEVs. Moreover, cluster 13 was predicted to be the capsule, as these spots preferred to appear at the boundaries of the lymph nodes. Finally, cluster 14 was suggested to be a tissue type outside of the lymph nodes with relatively high scores of plasma B cells. In summary, classical AITL histopathological patterns were observed in our ST landscape of AITL, including diverse proportions of neoplastic TFH-phenotype cells interspersed with FDCs, B lymphocytes, plasma cells, and HEVs [29] (Fig. 2G). The annotations of all the clusters were labeled and projected onto the UMAP and H&E-stained sections (Fig. 2B and Fig. S1B).

The consistency between the ST-SEQ and immunostaining data annotations was investigated. ST-SEQ revealed that the spots from AITL1 had an extremely high proportion of GC-related cluster 6, which was consistent with the preservation of follicular dendritic cell networks in AITL1 observed by immunostaining. Additionally, the neoplastic spots of AITL2 predominantly consisted of spots from cluster 1, which had higher proportions of macrophages and monocytes rather than B cells and FDCs. This finding was consistent with the negative results of CD10 and CD21 immunostaining for AITL2 (Fig. 1A and Fig. S1B). In summary, the immunostaining results and ST clusters were highly consistent with and supported each other.

### Exacerbation of inflammation and immune dysregulation in the neoplastic TME in AITL

**Fig. 3:**
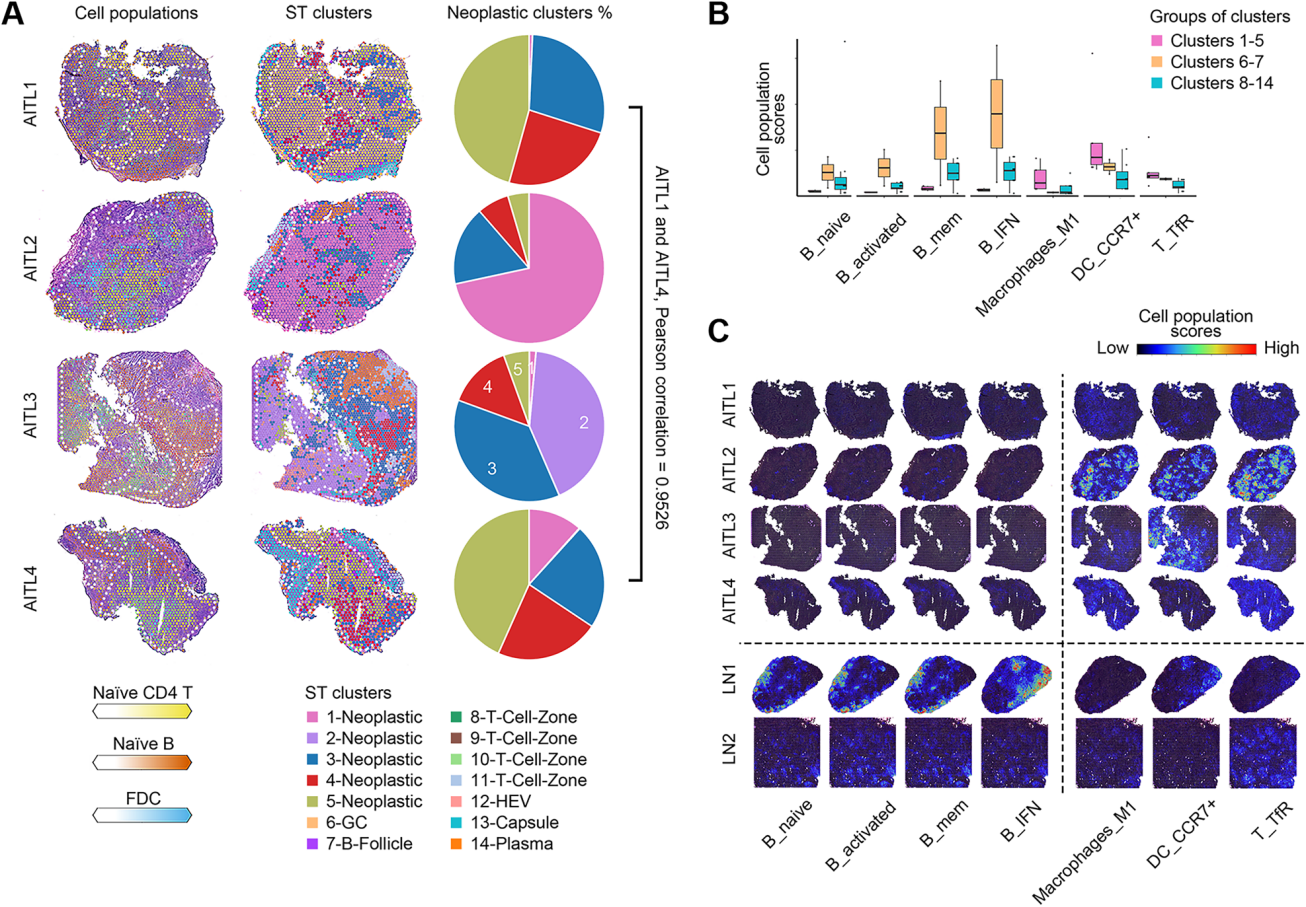
Exacerbation of inflammation and immune dysregulation in the neoplastic TME in AITL. **A** Predicted abundances of cell populations, ST cluster labels, and proportions of neoplastic clusters in AITL. The neoplastic spots are surrounded by white dashed lines. **B** Boxplots of the abundances of cell populations with significantly distinct scores between neoplastic clusters 1-5 and other clusters 6-14. **C** Frozen sections labeled by the abundances of cell populations with significantly lower (columns 1-4) or higher scores (columns 5-7) in the neoplastic TME. The scale of the cell population scores was consistent within each cell type. B_IFN, IFN-response B cell; B_activated, activated B cell; B_mem, memory B cell; B_naive, naïve B cell; DC_CCR7+, activated dendritic cells expressing CCR7; Macrophages_M1, M1 macrophages; T_TfR, T follicular regulatory cell.

To investigate the heterogeneity of the neoplastic TME in AITL, the abundances of naïve CD4 T cells, naïve B cells, and FDCs were projected to the frozen sections of AITL with H&E staining, which represented GCs, B follicles, and T-cell zones, respectively. The composition of these three cell types varied among different AITL samples. Moreover, the composition of clusters in the neoplastic TME was also estimated. AITL1 and AITL4 shared a similar composition of neoplastic clusters (Pearson correlation = 0.9526) but differed from the other two cases (Fig. 3A). In summary, the neoplastic TME showed high heterogeneity among AITL samples in terms of the compositions of both cell types and neoplastic clusters.

The difference in cell type abundance was further investigated to distinguish the neoplastic TME from the nonneoplastic TME. The neoplastic clusters had a significantly lower proportion of naïve, activated, memory, and IFN-responsive B cells, suggesting depletion of the follicle structure and verifying the global shift in B-cell phenotypes in AITL [24]. Conversely, neoplastic clusters showed significant enrichment of M1 macrophages, activated dendritic cells expressing *CCR7* (*CCR7*^+^ DCs), and T follicular regulatory cells (Tfr) (P < 0.05 and adjusted *P* < 0.25). Figure 3B–C). M1 macrophages are a group of polarized cells with proinflammatory activation [30]. *CCR7*^+^ DCs can migrate to T-cell zones in lymph nodes to promote antigen presentation and the T-cell response, leading to a series of inflammatory responses [31]. Tfr cells regulate both B cells and TFH cells in GCs to control B-cell autoreactivity [32, 33]. This observation demonstrated that the neoplastic TME in AITL exacerbated features of inflammation and immune dysregulation.

### An uncommon TME type lacking CD40-CD40LG activity and enriched with TAMs in AITL

**Fig. 4:**
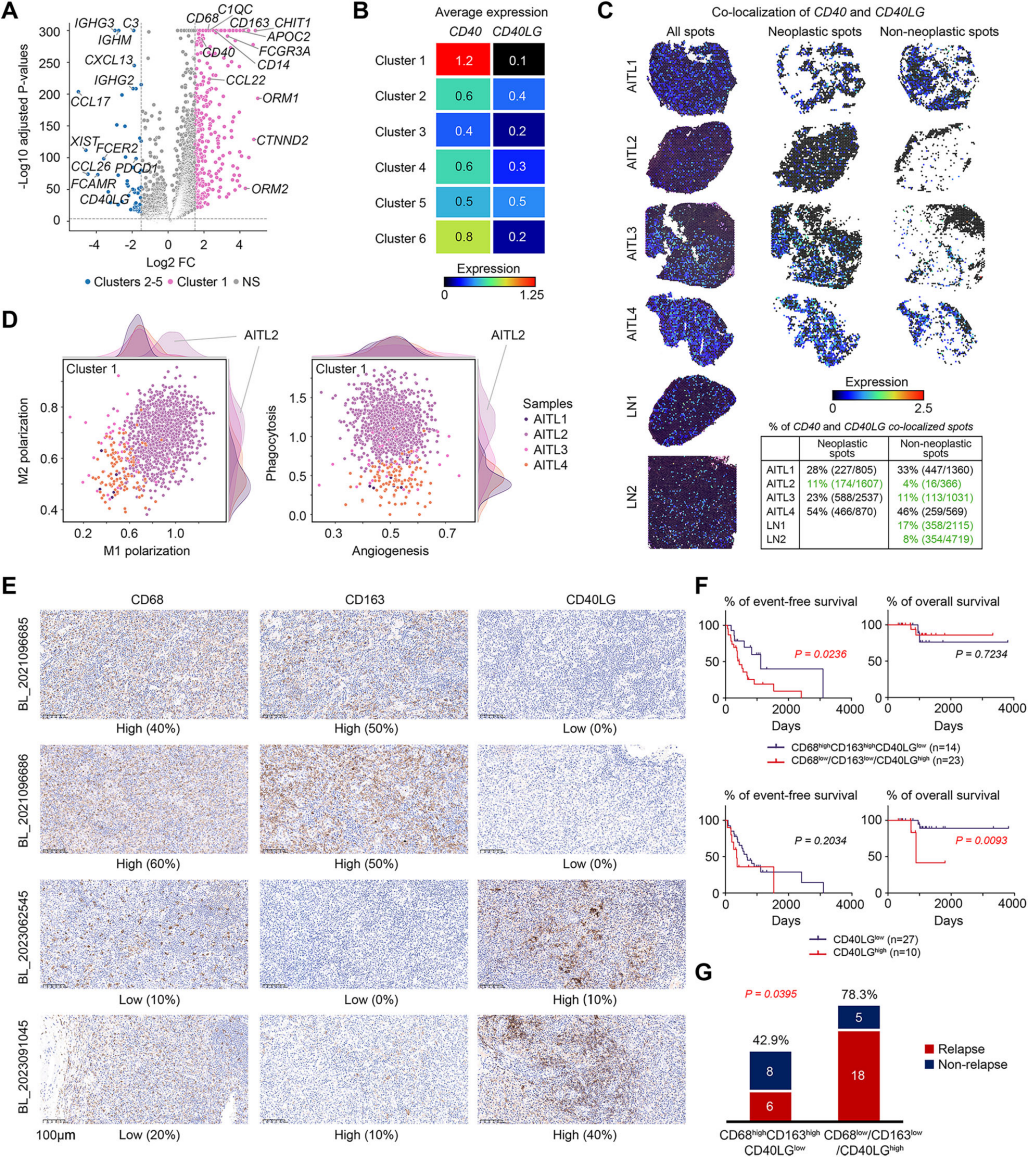
Molecular features and prognostic values of the uncommon neoplastic TME in AITL. **A** Volcano plot of highly expressed genes in cluster 1 vs. clusters 2-5. **B** Gene expression of *CD40* and *CD40LG* in clusters 1-6. **C** Colocalization of *CD40* and *CD40LG* at the ST spots. The colors of the spots represent the minimum expression values of *CD40* and *CD40LG*. **D** Expression of M1 vs. M2 polarization signatures and angiogenesis vs. phagocytosis signatures in spots from cluster 1. **E** Representative sections from the AITL validation cohort stained for CD68, CD163, and CD40LG. Each row represents a sample. BL in the sample ID is short for the baseline. **F** Survival analysis of the validation cohort divided by either CD68^high^CD163^high^CD40LG^low^ or CD40LG expression. **G** Numbers and proportions of patients who experienced relapse among patients with AITLs with CD68^high^CD163^high^CD40LG^low^ or CD68^low^/CD163^low^/CD40LG^high^ TME types.

The top expression patterns of cluster 1 were notably distinct from those of neoplastic clusters 2-5, as shown in Fig. 2D, indicating the presence of two distinct types of neoplastic TMEs in AITL. To decipher the characteristics of these two types, the DEGs of neoplastic cluster 1 were compared with those of clusters 2-5 (Fig. 4A and Table S7). Clusters 2-5 highly expressed Ig genes and Fc receptor genes, reflecting a B-cell-enriched TME. Additionally, chemokine genes, including *CCL17*, which was highlighted in an ST-sequenced case report and putatively associated with immune suppression in AITL, were highly expressed in this TME [25]. This finding was also supported by the high expression of *PDCD1* in these neoplastic TMEs. In contrast, cluster 1 highly expressed genes associated with myeloid cells, including *CD68*, *CD163*, *C1QC*, *CD14*, and *FCGR3A* (which encodes CD16), indicating increased proportions of macrophages and monocytes.

Clonal GC B cells function as a niche for AITL, supporting tumorigenesis via a stronger *CD40‒CD40LG* axis. A recent study revealed significantly prolonged survival in AITL model mice treated with anti-Cd40lg inhibitory antibodies [23]. Interestingly, *CD40* and *CD40LG* were highly expressed in clusters 1 and 2-5, respectively (Fig. 4A). Thus, the two types of neoplastic TME potentially predict different responses to anti-CD40LG therapy. *CD40* was highly expressed in cluster 1 and GC cluster 6 and moderately expressed in clusters 2-5. Conversely, *CD40LG* was rarely expressed in cluster 1 and, as expected, was expressed at higher levels in both clusters 2-5 than in cluster 6 (Fig. 4B). To investigate the inconsistent activity of the counterparts, we defined the activity of the *CD40*-*CD40LG* axis by coexpression of the ligand and receptor in the same spot quantified by the lower expression level. Three of the four AITLs showed stronger *CD40*-*CD40LG* activity than the LN sections. AITL2, which predominantly consisted of spots from cluster 1, showed notably weaker *CD40*-*CD40LG* activity than the other AITLs and LNs in both neoplastic (11% vs. 23–54%) and nonneoplastic (4% vs. 11–46%) TMEs (Fig. 4C). Considering the conventional features of the AITL TME with strong *CD40*-*CD40LG* activity [23, 25], AITL2 represented an uncommon TME type in AITL, whose tumorigenesis was weakly supported by GC B cells via the *CD40*-*CD40LG* axis.

The uncommon TME type is enriched with TAMs, which have highly variable functions across different types of cancers [34]. To decipher the functions of TAMs in the uncommon TME of AITL2, we investigated the M1/M2 dualistic polarization, as well as the angiogenesis/phagocytosis dichotomous functional phenotypes of TAMs [30, 35] in all spots from cluster 1, using the gene sets employed in our previous study [18]. The cluster 1 spots from AITL2 coexpressed both higher M1 and M2 signatures. TAMs with this expression pattern are widely observed in many types of cancers [34]. When TAMs in lymphoma were examined in the study from Cheng S et al., only *ISG15*^+^ and *C1QC*^+^ TAMs exhibited this pattern, and only the latter gene was significantly highly expressed in cluster 1 spots (Fig. 4A). Furthermore, the spots from AITL2 presented increased expression levels of the phagocytosis signature, with similar angiogenesis functions as those from AITL1, AITL3, and AITL4. This finding was consistent with *C1QC*^+^ TAMs preferentially expressing genes involved in phagocytosis and antigen presentation [35] (Fig. 4D). Thus, the uncommon AITL TME highly exhibited signatures of both M1 and M2 macrophages and is associated with phagocytosis-associated *C1QC*^+^ TAMs.

### The uncommon TME predicted longer EFS and less relapse in AITL patients

The uncommon AITL TME showed high expression levels of *CD68* and *CD163*, indicating an increased proportion of macrophages (Fig. 4A). It also lacked the conventional features of *CD40LG* expression, suggesting that it is weakly supported by GC B cells via the CD40-CD40LG axis (Fig. 4B–C). To validate the prognostic value of the uncommon TME type, three markers —CD68, CD163, and CD40LG —were used to stain 37 newly diagnosed AITLs for sample stratification. A histological feature of CD68^high^CD163^high^CD40LG^low^ was used to represent the uncommon TME type, which lacked *CD40*-*CD40LG* activity and exhibited highly expressed TAM signatures (Fig. 4E). Among this validation cohort, 25 patients were male, and 12 patients were female. The median age was 62 years (range 38-77). All patients accepted the CHOP-based regimen as the first-line therapy. The median follow-up duration was 998 days (range 329–3808 days). The last follow-up date was 30/5/2024. The baseline information for the patients in the validation cohort is presented in Table S8. A CD68^high^CD163^high^CD40LG^low^ TME was associated with longer EFS (*P* = 0.0236) and significantly less relapse (*P* = 0.0395). To confirm CD68^high^CD163^high^CD40LG^low^ TME as an independent prognostic factor, adjusting for potential confounding factors, multivariate Cox regression analysis was performed, revealing a significant (*P* = 0.014) eight times longer EFS than the other patients (Table S9). Additionally, lower expression of the CD40LG protein was associated with longer OS (*P* = 0.0093) (Fig. 4F, G).

### The uncommon TME type presents a distinct molecular evolutionary trajectory in AITL

**Fig. 5:**
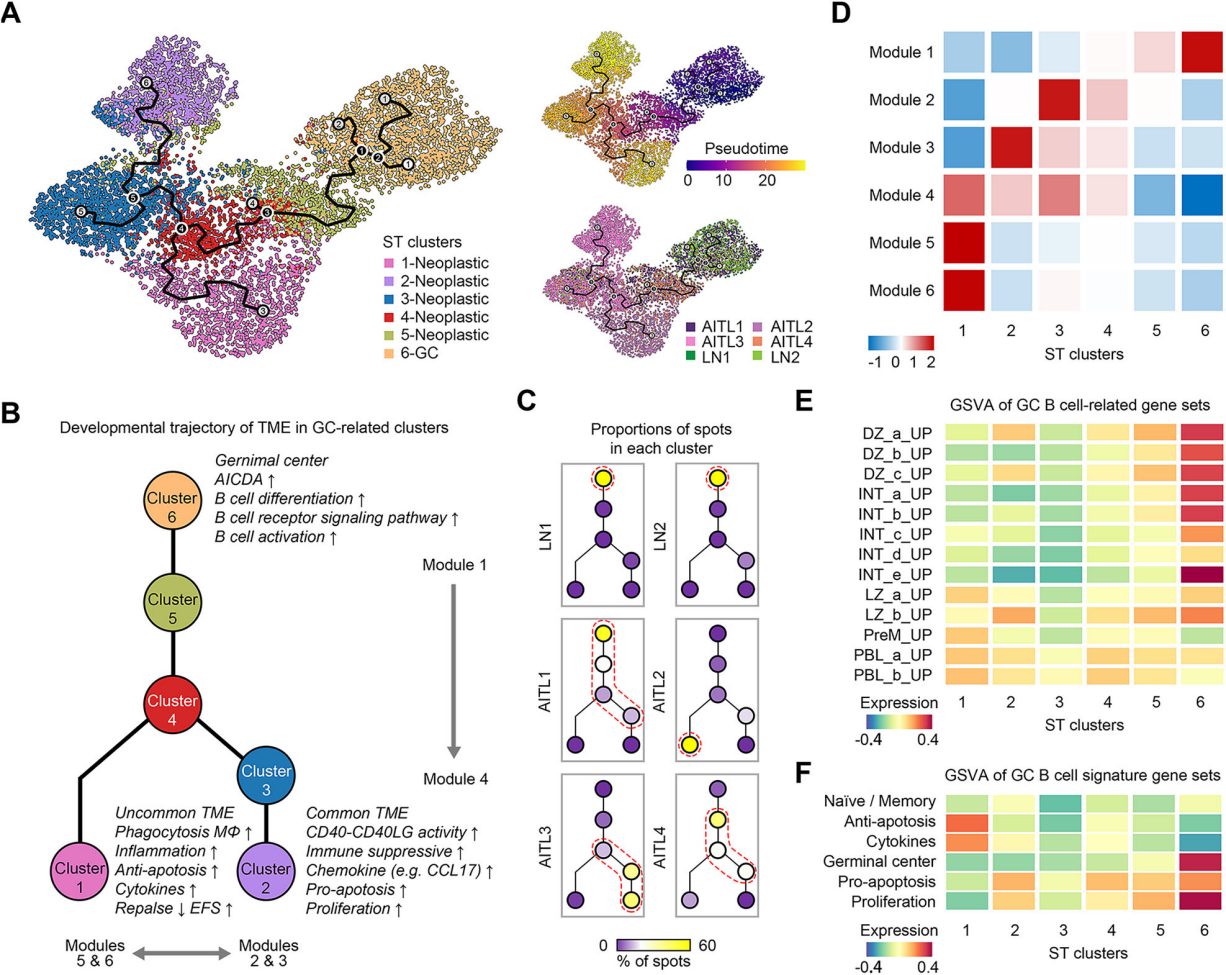
Trajectory analysis of spots from GC-related clusters. **A** Trajectory of spots from GC-related clusters, which were labeled by cluster annotations, pseudotime, and sample sources. **B** Bifurcated model of the histological evolution of the TME in AITL. **C** Distribution of spots at each node of the model for samples. **D** Significant gene modules identified via autocorrelation analysis and their changes across the trajectory. GSVA of spots from GC-related clusters via (**E**) GC B-cell-related and (**F**) B-cell signature gene sets. DZ, dark zone; INT, transitional profiles between dark zone and light zone; LZ, light zone; PreM, precursor memory B cell, and PBL, plasmablast.

To elucidate whether the uncommon TME type represented a distinct entity or an intermediate state of the common TME type during AITL neoplasia, we conducted a pseudotime analysis to determine if cluster 1 developed independently from other neoplastic clusters. GC cluster 6 was incorporated to trace the origin of TME development. Clusters 1-6 were reclustered to construct the trajectory of molecular evolution and identify the genes driving this process (Fig. 5A). Pseudotime trajectory analysis proposed a bifurcated model for the histological evolution of the TME in AITL patients (Fig. 5B). The spots from both LNs showed similar distributions and converged at the root cluster, whereas the spots from AITLs diverged into separate branches A continuous shift in the spot distribution was observed, progressing from AITL1 to AITL4 and to AITL3. The proximity of spots from AITL1 and AITL4 on the model corroborated the similar composition of neoplastic clusters in these two samples. In contrast, the spots from AITL2 were located in distinct branches, confirming that AITL2 represented an uncommon and distinct neoplastic TME compared to the conventional group of AITL (Fig. 5C).

To explore the functions of genes driving this trajectory, we identified gene modules that were significant in the autocorrelation analysis and demonstrated changes across the developmental trajectory (Fig. 5D and Table S10). Module 1 showed the highest scores in cluster 6, reflecting the origin of the GC pattern. This module consisted of genes such as *MS4A1*, *CD79A/B*, *PAX5*, and *AICDA*. It was significantly enriched in biological processes related to B-cell differentiation (FDR = 0.0002) and activation (FDR = 0.0243), the B-cell receptor signaling pathway (FDR = 0.0004), and the GC phenotype. The trajectory of the common group of AITLs was represented in an orderly manner by modules 2 and 3. Module 2 comprised immunoglobulin genes, especially the IgG genes generated through the class-switching process. Module 3 expressed *CR2*, *FCAMR*, and *FCER2*, which facilitated the binding of immunoglobulins. In contrast, the trajectory of the uncommon TME in AITL2 was represented by both modules 5 and 6. Genes within these modules were significantly enriched in the biological processes of the inflammatory response (FDR = 0.0064) and the Fc-gamma receptor signaling pathway (FDR = 0.0068), confirming their association with TAM (Table S11).

### The uncommon TME consists of GC B cells associated with antiapoptotic effects and cytokines

Given that GC B cells serve as niches supporting AITL tumorigenesis, we investigated the evolution of GC B cells in the TME of AITL and LN patients, focusing on GC-related clusters 1-6. Gene set variable analysis (GSVA) [36] was performed via the gene sets related to GC B cells [37] or from Chung and colleagues [38]. Compared to cells from the GC cluster, B cells from all of the neoplastic tissues presented a more differentiated phenotype, with enrichment of light zones, prememory B cells, and plasmablasts (Fig. 5E). Spots from both neoplastic clusters 2-5 and GC cluster 6 presented higher scores for pro-apoptosis and proliferation, with the highest scores in the GC cluster and the lowest scores in the neoplastic clusters. However, spots from neoplastic cluster 1 demonstrated the highest scores for antiapoptotic functions and cytokines, indicating a distinctive phenotype of the uncommon TME in AITL patients (Fig. 5F). In summary, we have identified an uncommon TME type in AITL, characterized by CD40-CD40LG activity deficiency and TAM enrichment. This TME type was associated with functions of the inflammatory response, antiapoptotic effects, and cytokines and predicted less relapse and longer EFS (Fig. 5B).

## Discussion

In this study, we constructed a comprehensive ST landscape of AITL to identify 14 distinct clusters, including five neoplastic clusters, which revealed exacerbated inflammatory responses and immune dysregulation within the neoplastic TME. Notably, we characterized a rare TME phenotype in AITL marked by absent *CD40*-*CD40LG* activity and enrichment of TAMs. Validation in an expanded AITL cohort confirmed that this TME subtype was associated with reduced relapse rates and prolonged EFS. Trajectory analysis further revealed a distinct molecular evolution in this TME phenotype, characterized by an enhanced inflammatory response, antiapoptotic effects, and cytokine dysregulation.

An expression signature-based prognostic model for AITL was previously delineated, highlighting the association between monocytic gene signatures and poor prognosis [39]. In our study, AITL cases with a CD68^high^CD163^high^CD40LG^low^ TME exhibited prolonged EFS and similar OS compared to other groups. However, CD68 or CD163 expression alone or their combination did not yield significant differences in EFS or OS. This discrepancy might stem from the phenotypic complexity of TAM in AITL [34], which could be obscured by bulk RNA-SEQ that averages signals from millions of cells. TAM-associated neoplastic spots (predominantly from Cluster 1) were observed in all four AITL cases in the discovery cohort. However, spots from AITL2 displayed divergent expression profiles of M1/M2 polarization and phagocytosis signatures relative to other samples. This underscores the necessity of high-resolution methodologies, particularly scRNA-SEQ and ST-SEQ, for deciphering the TME heterogeneity of AITL.

The AITL TME is uniquely heterogeneous and intimately linked to tumorigenesis and patient prognosis, driving efforts to develop TME-targeted immunotherapies. While PD-1 serves as a diagnostic biomarker for AITL, a clinical trial of PD1-PDL1 blockade in peripheral T-cell lymphomas (including AITL) was terminated due to high rates of hyperprogression, modest efficacy, and short response durations [40]. This finding suggested that PD1-PDL1 signaling may exert suppressive rather than promotive effects on AITL neoplastic cells [41, 42]. Conversely, recent work has demonstrated that GC B cells support AITL lymphomagenesis via the *CD40-CD40LG* axis, with anti-CD40LG antibody treatment prolonging survival in AITL mouse models [23]. These findings provide new options for targeted immunotherapy for AITL. Notably, the rare TME subtype identified in our cohort lacked *CD40-CD40LG* activity, emphasizing the critical need for TME-based disease stratification in AITL.

In summary, our study established a comprehensive ST landscape of AITL, deciphering TME heterogeneity and subtypes with implications for targeted immunotherapy. We demonstrated the prognostic significance of neoplastic TME stratification, although our analysis was constrained by the inherent heterogeneity of neoplastic/non-neoplastic cell populations and limited patient sample size. While validation in an expanded cohort supported our key findings, further characterization of rare AITL TME subtypes will be essential to facilitate precise patient stratification and optimize immunotherapeutic strategies.

## Materials and methods

### Patients and samples

A total of 41 patients who had adequate neoplastic tissue biopsies with a confirmed diagnosis of AITL were recruited. For the discovery cohort, four AITL samples and two inflamed lymph node control samples from noncancerous donors were collected. For the validation cohort, 37 newly diagnosed AITL patients were retrospectively enrolled from 24/12/2013 to 26/6/2023 at The First Affiliated Hospital to Zhejiang University School of Medicine. The diagnosis of AITL relies on lymphadenectomy biopsy, and the pathological criteria for AITL refer to the World Health Organization classifications [14]. The diagnosis of AITL was confirmed by two different pathological experts. Therapeutic strategies and response evaluations for AITL patients were performed according to the National Comprehensive Cancer Network (NCCN) guidelines for T-cell lymphomas [43]. EFS was defined as the duration from the date of diagnosis to the date of refractoriness, relapse, or death. OS was defined as the duration from the date of diagnosis to the date of death. Informed consent was obtained from all patients or donors. The institutional review board at The First Affiliated Hospital to Zhejiang University School of Medicine approved the study, with approval number IIT20240804A.

### WES and data processing

Genomic DNA was extracted from formalin-fixed, paraffin-embedded (FFPE) biopsies derived from AITL patients via the DNeasy Blood & Tissue Mini Kit (Qiagen) following the manufacturer’s instructions. WES was performed via Illumina’s NovaSeq platform. The sequencing reads containing adapter sequences, low-quality reads (no-call positions >10%), and low-quality bases ( > 50% bases with quality <5) were removed. High-quality paired-end reads were aligned to the University of California Santa Cruz (UCSC) human reference genome (hg38) via BWA [44]. Germline single-nucleotide polymorphisms (SNPs) and insertions and deletions (indels) were identified via the HaplotypeCaller subtool in GATK [45]. Because there were no control samples for the tumor biopsies, putative tumor-only SNVs and indels were identified by filtering germline SNPs and indels as follows. First, the SNPs and indels localized in intronic regions or annotated as synonymous variants were filtered out. Second, if an SNP or indel was further indicated as “benign” or “likely benign” in ClinVar [46], the variant was filtered out. Third, because of the rare tumor content of AITL, variants with a variant allele frequency (VAF) greater than 90% were removed as homozygous. Next, the variants with frequencies greater than 1% in the population database of the Exome Aggregation Consortium (ExAC) were removed [47]. Finally, the variants not belonging to the frequently mutated AITL genes [27] were also removed.

### Spatial transcriptome sequencing of frozen tissues

Fresh tissues from the 4 AITLs and 2 lymph node controls were collected and embedded in a cryomold (Shitai, catalog no. 80203-0007) by optimal cutting temperature (OCT, Sakura, catalog no. 4583) compound on dry ice and stored at −80 °C. The frozen OCT-embedded tissues were cut into 10 μm sections. The cut sections were placed in capture areas of Visium spatial slides (10x Genomics, catalog no. PN-1000184). The slides were permeabilized for 12 min according to the Visium Spatial Tissue Optimization protocol (10x Genomics, catalog no. CG000238). Images of the stained slides were acquired via the Nikon Eclipse Ti2 system. Finally, the libraries were constructed following the Visium Spatial Gene Expression protocol (10x Genomics, catalog no. CG000239), and PE150 was sequenced on the Illumina platform.

### Data processing of ST-SEQ data

The sequencing data were first preprocessed via Space Ranger v1.3.1 and subsequently aligned to the GRCh38 reference genome. The spots with UMI counts less than 500, fewer than 500 genes, and a percentage of mitochondrial counts greater than 5% were filtered out. The remaining data were then normalized with Scanpy (version 1.9.1) [48]. To remove batch effects, Harmony was performed on the basis of the top 5000 highly variable genes [49]. Dimension reduction was performed via principal component analysis (PCA). The top 24 principal components were then summarized via UMAP to project data in a two-dimensional panel. Finally, clusters were identified via the Leiden algorithm [50].

### Projection of the cell population to ST spots

The reference cell populations of human secondary lymphoid organs were constructed by citing Kleshchevnikov et al.’s integration of three studies [51–53]. Cell2location, a Bayesian model that estimates the absolute abundance of cell types at each spot, was then used to map the reference cell populations to our ST spots via default parameters [28]. First, the regression model for the single-cell data was initialized with default settings, using batch as the batch_key. Next, the regression model for the spatial transcriptomics data was initialized with the single-cell reference signatures, default settings, and hyperparameters selected on the basis of the cell2 location recommendations. We did not observe strong within-batch variation in the total RNA count; therefore, we set detection_alpha = 20. By manual visual inspection of a subset of spots, we estimated the approximate mean number of cells per spot to be 10 and therefore set N_cells_per_location = 10. The model was then trained via a maximum of 20,000 epochs. Finally, the estimated mean abundances of the cell types were rounded, and spots with no estimated cells were assigned one cell to the cell type with the greatest abundance.

### Identification of differentially expressed genes

The differentially expressed genes (DEGs) were identified via the Mann–Whitney *U* test and defined as having an average expression in one ST cluster that was >2^0.5^-fold higher than that in the other ST clusters and were required to have a detectable expression in 25% of all spots in that ST cluster and had an adjusted *p*-value (*q* value) < 0.0001.

### Trajectory analysis

Pseudotime trajectory analysis of the neoplastic and GC clusters was performed via Monocle 3 [54]. The top 2000 variable genes from the Seurat object were loaded to Monocle 3. The trajectory analysis was performed via the learn_graph function.

### Immunohistochemistry staining

Immunohistochemical analysis was performed on formalin-fixed, paraffin-embedded tissue sections. Briefly, after deparaffinization in xylene and rehydration in graded alcohols, endogenous peroxidase was blocked with 3% hydrogen peroxide. Antigen retrieval was performed via citrate buffer or protease digestion, as appropriate for each antibody. After rinsing in phosphate-buffered saline, antibodies specific for CD68 (ZSGB-BiO, ZM-0464, 1:200), CD163 (ZSGB-BiO, ZM-0428, 1:300) and CD40LG (Abcam, ab303610, 1:500) were used for immunohistochemical staining. These stains were performed via an automated immunostainer (Benchmark XT System; Ventana Medical System, Tucson, AZ, USA) and a streptavidin‒biotin peroxidase detection system. The slides with immunoreactivity were scored in at least three randomly selected high-power fields. All the sections were scored by two experienced pathologists.

### Statistics

Statistical analysis was performed via the Mann–Whitney *U* test. Significance was determined at *p* < 0.05. Multiple test correction was performed using the false discovery rate (FDR). Survival analysis was performed via the Kaplan‒Meier method and tested via the log-rank test.

## Supplementary information

Supplemental Figures 1-2

Supplemental Tables 1-11

## Data Availability

The datasets generated during and/or analyzed during the current study are available from the corresponding author upon reasonable request. The raw sequence data reported in this paper have been deposited in the Genome Sequence Archive in National Genomics Data Center, China National Center for Bioinformation/Beijing Institute of Genomics, Chinese Academy of Sciences (GSA-Human: HRA011643 [WES], HRA011659 [ST-seq]) that are publicly accessible at https://ngdc.cncb.ac.cn/gsa-human.
