# Supplementary material for: Revealing tumor microenvironmental heterogeneity and prognostic value in angioimmunoblastic T-cell lymphoma via spatial transcriptome sequencing
Source: Cell Death Dis. 2026 Jan 9;17(1):27. doi: 10.1038/s41419-025-08212-9 (PMC12789568; doi:10.1038/s41419-025-08212-9)
Supplement: Supplementary file 1 — Supplemental Figures 1-2 [file 41419_2025_8212_MOESM1_ESM.docx]

**Supplemental Figures 1-2**

**
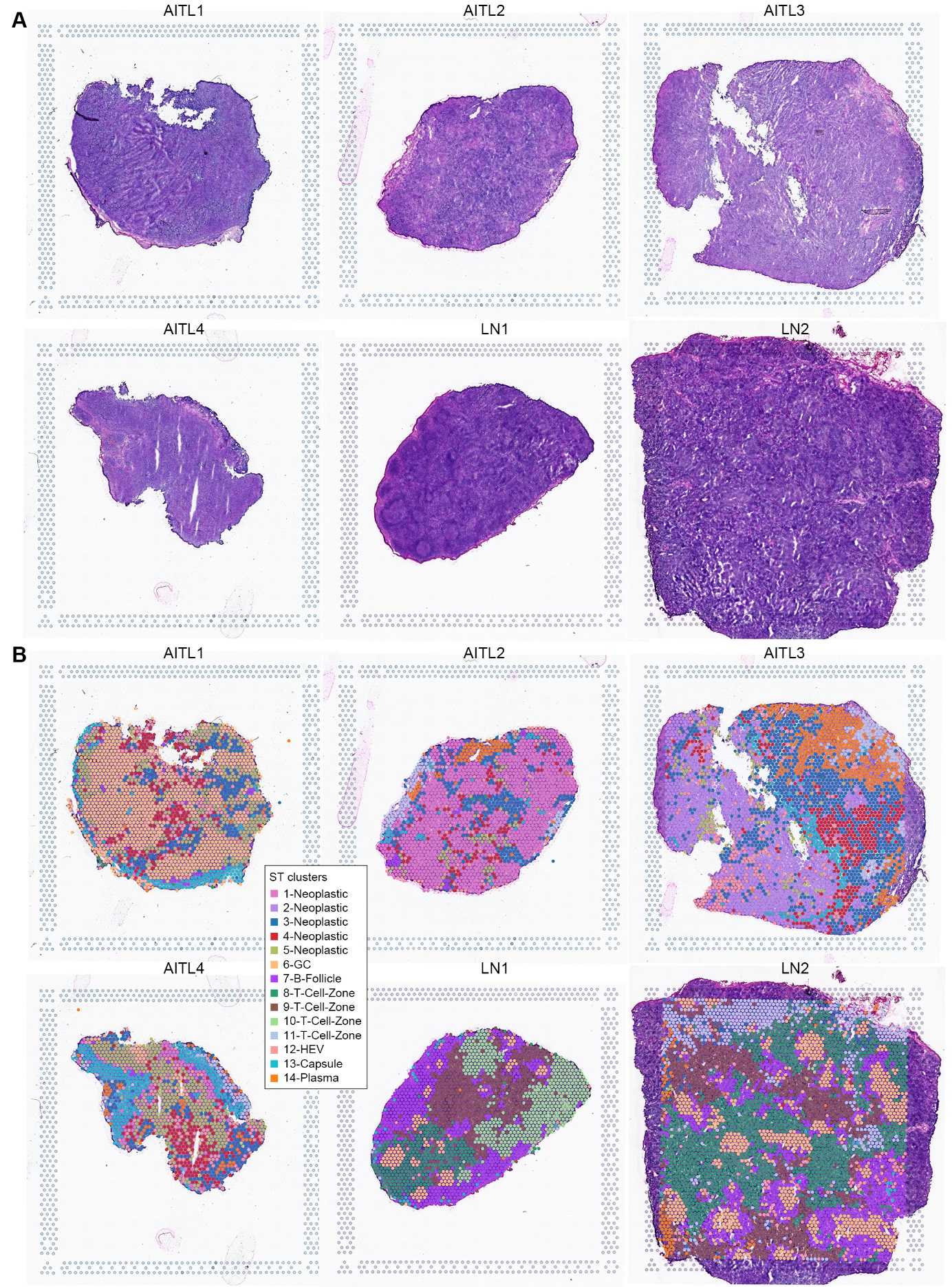
**

**Figure S1. H&E staining and cluster projection of** **frozen sections used for ST-SEQ.** (A) H&E staining of 4 AITLs and 2 LNs. (B) Projecting the ST clusters on the frozen sections. The annotations of the clusters were the same as those in Figure 2B.


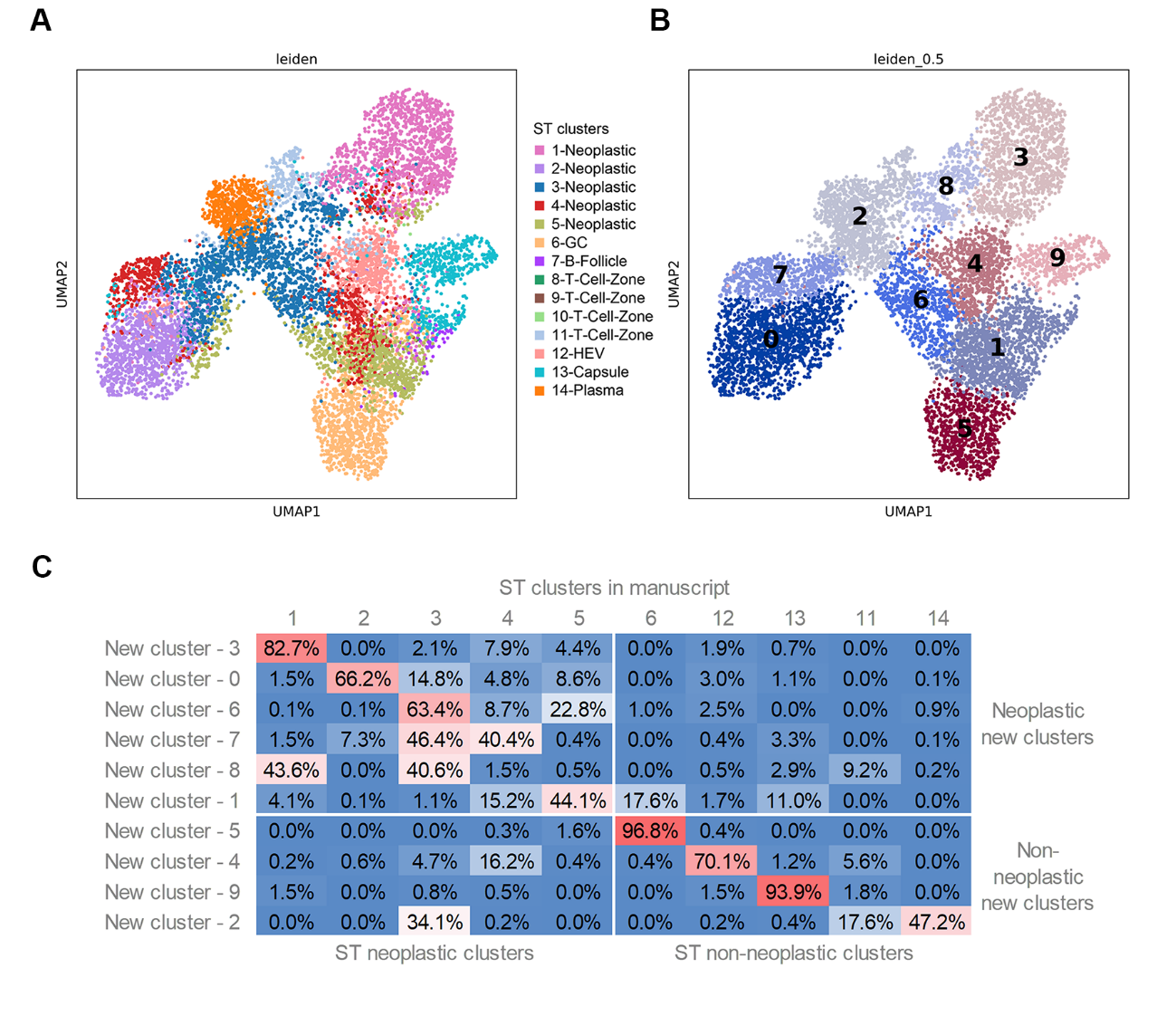


**Figure S2. The reclustering analysis with only AITL spots.** The reclustering was performed only using the spots from AITLs with (A) labeling using the ST clusters with current annotation in Figure 2B or (B) labeling using clusters newly generated in the reclustering. (C) The proportions of neoplastic spots (ST clusters 1-5) or nonneoplastic spots (ST clusters 6-14) in each new cluster are shown. ST clusters 7-10 were not included because each of them contained no more than 100 spots. The percentages were calculated within each new cluster.
